# Performance evaluation of the Molbio diagnostics Truenat MTB Ultima/COVID-19 multiplex assay for TB and COVID-19 case detection among people with symptoms suggestive of tuberculosis—a study protocol for clinical trials

**DOI:** 10.3389/fpubh.2025.1620210

**Published:** 2025-06-27

**Authors:** Manju Purohit, Willy Ssengooba, Helen Cox, Cesar Ugarte-Gil, James Sserubiri, Hafsah Tootla, Kavindhran Velen, Rita Székely, Adam Penn-Nicholson

**Affiliations:** ^1^Department of Pathology, R D Gardi Medical College, Ujjain, MP, India; ^2^Department of Public Health Sciences, Karolinska Institutet, Stockholm, Sweden; ^3^Department of Medical Microbiology, Makerere University, Kampala, Uganda; ^4^Division of Medical Microbiology, Faculty of Health Sciences, University of Cape Town, Cape Town, South Africa; ^5^Department of Epidemiology, School of Public and Population Health, The University of Texas Medical Branch, Galveston, TX, United States; ^6^Instituto de Medicina Tropical Alexander von Humboldt, Universidad Peruana Cayetano Heredia, Lima, Peru; ^7^National Health Laboratory Service, Department of Medical Microbiology, Red Cross War Memorial Children’s Hospital, Cape Town, South Africa; ^8^FIND, Geneva, Switzerland

**Keywords:** Truenat COMBO, tuberculosis, COVID-19, diagnosis, protocol

## Abstract

**Background:**

Tuberculosis (TB) remains a major public health problem globally, as reflected in persistently high morbidity and mortality rates. Current control efforts have been further complicated by the ongoing SARS-CoV-2 (COVID-19) pandemic. Rapid molecular diagnostics remain crucial to identifying the millions of people with undiagnosed TB.

**Objectives:**

The study aimed to determine the diagnostic accuracy of Truenat MTB Ultima/COVID-19 for the detection of COVID-19 and TB among presumptive TB participants, using a microbiological reference standard (MRS) and a country-approved real-time reverse transcription polymerase chain reaction (RT-PCR) COVID-19 assay.

**Methods:**

This prospective cohort study assessed the diagnostic accuracy of the Truenat MTB Ultima/COVID-19 multiplex test among adults with presumptive TB, enrolled from healthcare facilities in four countries, aiming to reach 270 confirmed TB cases. The inclusion criteria were as follows: adults who self-reported at least one symptom suggestive of pulmonary TB, were willing to return for a day 2 visit, and agreed to provide oral swab samples for bio-banking. Patients were excluded from enrolment in the study if they had started anti-TB treatment within 60 days or TB preventive therapy within 6 months prior to enrolment or if they were unable to provide 3 mL of sputum or nasopharyngeal (NP) and tongue swab samples.

**Expected outcome:**

This study expected to obtain point estimates of the sensitivity and specificity of the Truenat MTB Ultima/COVID-19 multiplex test for TB and COVID-19 detection, compared to Xpert Ultra, among presumptive TB participants using an MRS and pre-defined COVID-19 assay.

## Introduction

Tuberculosis (TB) remains a major public health problem globally, as reflected in persistently high morbidity and mortality rates (1). TB and coronavirus disease 2019 (COVID-19) are infectious diseases that primarily affect the lungs and present with overlapping clinical symptoms, radiological findings, and laboratory features, making them difficult to distinguish (2, 3). People and communities vulnerable to TB are also likely to be at a greater risk of COVID-19 infection due to shared risk factors, such as crowded living conditions and co-morbidities such as malnutrition and diabetes (3). Co-infection with SARS-CoV-2 and TB is also associated with poorer outcomes. Studies from South Africa and the Philippines indicate that people with TB appear to be at a substantially greater risk of death from COVID-19 than those without TB (4, 5). Similarly, studies and comprehensive meta-analyses have revealed that co-infection is consistently associated with worse clinical outcomes, including increased mortality and prolonged recovery periods (6–8). A further concern is that reduced immunity and lung inflammation caused by COVID-19 and the corticosteroid therapy used to treat COVID-19 may lead to the progression of TB infection to symptomatic disease or worsening of active TB (9). During the COVID-19 pandemic, there was a decrease in TB case detection in many settings (10–12). The COVID-19 pandemic indirectly impacted TB outcomes by straining healthcare systems and causing service disruptions. These findings emphasize the importance of integrated diagnostic strategies and continuous care for TB patients.

Globally, there have been calls for integrating TB and COVID-19 testing, given their shared respiratory manifestation and the need for rapid diagnosis to limit onward transmission and reduce associated morbidity and mortality. This approach involves screening all COVID-19 patients for TB symptoms, and vice versa, particularly in countries with a high burden of TB. However, in low-resource settings, diagnostic capacity remains a critical bottleneck. Integrating TB and COVID-19 testing platforms, expanding mobile diagnostic services, and training healthcare personnel to recognize co-infection scenarios are essential for improving outcomes.

Molecular diagnostics remain central to confirming the presence of both infections. COVID-19 is diagnosed primarily through reverse transcription polymerase chain reaction (RT-PCR) or rapid antigen testing from nasopharyngeal (NP) or oropharyngeal swabs. In contrast, TB diagnosis requires sputum smear microscopy, culture, or nucleic acid amplification tests such as the Xpert MTB/RIF Ultra assay or line-probe assays. These tests not only confirm TB but can also identify rifampicin resistance, which is critical for appropriate treatment planning. The use of oral and nasopharyngeal (NP) swabs became widespread with the advent of COVID-19 testing. Early evidence suggests that this sampling method may be additionally effective for TB detection (13, 14). In people with pulmonary TB, *Mycobacterium tuberculosis* (MTB) deoxyribonucleic acid (DNA) is deposited in the oral cavity and can be detected through molecular diagnostics (15). A study using the same sputum sample for TB and COVID-19 diagnosis with the Xpert platform showed that diagnostic integration is feasible, but the performance is moderate (16). In particular tongue swabbing improves sampling efficiency (biomass collection) compared to buccal or other oral swabbing techniques (17). Identifying an efficient method that supports both TB and COVID-19 sampling from the same specimen would be even more advantageous (18). Molbio Diagnostics (Molbio, Goa, India) responded to this unmet need with the development of the Truenat™ MTB Ultima/COVID-19 multiplex assay, which is capable of diagnosing both diseases in a single test.

We designed the study, which, to the best of our knowledge, is the first such study aimed at evaluating the performance of the Truenat MTB Ultima/COVID-19 multiplex test using prospectively collected nasopharyngeal swabs and sputum samples from participants with symptoms suggestive of TB. As a secondary objective, we evaluated the diagnostic accuracy of the assay using easier-to-collect specimens of mid-turbinate nasal swabs mixed with oral tongue swabs. The study’s primary, secondary, and exploratory objectives and respective outcome measures as endpoints are described in Table 1. The data gathered from this study will form part of the dossier to be submitted to the WHO for review.

**Table 1:** Study objectives and respective endpoints.

Objectives	Endpoints
Primary	
To determine the diagnostic accuracy of the Truenat MTB Ultima/COVID-19 multiplex assay for TB detection among the presumptive TB patients using an MRS	Point estimates of the sensitivity and specificity of the Truenat MTB Ultima/COVID-19 assay, with 95% confidence intervals, using the defined TB MRS
Secondary	
To determine the diagnostic accuracy of Truenat MTB Ultima/COVID-19 assay for COVID-19 detection among the presumptive TB patients using a country-approved RT-PCR COVID-19 assay To determine the diagnostic accuracy of the Truenat MTB Ultima/COVID-19 multiplex assay for TB detection compared to Xpert Ultra among the presumptive TB patients using culture as the reference standard To determine the prevalence of COVID-19 (by RT-PCR) among the presumptive TB patients investigated for COVID-19 To determine the prevalence of COVID-19 (by RT-PCR) among the patients identified as having TB by MRS To assess the feasibility and ease of use of self-sampled swab	Point estimates of the sensitivity and specificity of the Truenat MTB Ultima/COVID-19 assay, with 95% confidence intervals, using a country-approved RT-PCR COVID-19 assay Point estimates of the sensitivity and specificity of the Truenat MTB Ultima/COVID-19 multiplex assay for TB detection compared to Xpert Ultra among the presumptive TB patients using culture as the reference standard Estimate of the proportion of presumptive TB patients with COVID-19, confirmed by a country-approved RT-PCR Covid-19 assay (expressed as a percentage) Estimate of the proportion of TB patients (confirmed by MRS) with COVID-19 (confirmed by a country-approved RT-PCR COVID-19 assay) (expressed as a percentage) Analysis of survey responses using proportions and Linkert scale averages
Tertiary/Exploratory	
To determine the diagnostic accuracy of the Truenat MTB Ultima/COVID-19 multiplex assay using a combination of tongue and nasal swab samples for TB detection among the presumptive TB patients using a microbiological reference standard (MRS) To determine the diagnostic accuracy of the Truenat MTB Ultima/COVID-19 assay using a combination of tongue and nasal swab samples for COVID-19 detection among the presumptive TB patients using a country-approved RT-PCR COVID-19 assay Provision of diabetes and hypertension screening To determine the diagnostic accuracy of the Truenat MTB Ultima/COVID-19 multiplex assay for COVID-19 detection among the biobanked NP and sputum samples, using a country-approved RT- PCR COVID-19 assay	Point estimates of the sensitivity and specificity of the Truenat MTB Ultima/COVID-19 assay using a combination of tongue and nasal swab samples, with 95% confidence intervals, using the defined TB MRS Point estimates of the sensitivity and specificity of the Truenat MTB Ultima/COVID-19 assay using a combination of tongue and nasal swab samples, with 95% confidence intervals, using a country-approved RT-PCR COVID-19 assay Proportion of participants with elevated blood glucose levels (GLs) (random or fasting) among those who were not previously diagnosed with diabetes Point estimates of the sensitivity and specificity of the Truenat MTB Ultima/COVID-19 assay among the biobanked NP and sputum samples, with 95% confidence intervals, using a country-approved RT- PCR COVID-19 assay

## Methods

### Study design

To the best of our knowledge, this multicenter cohort clinical trial represents the first large-scale evaluation of prospectively collected fresh NP swabs and sputum samples to assess the accuracy of the Truenat MTB Ultima/COVID-19 multiplex assay used for the detection of TB and COVID-19 in people presenting with symptoms suggestive of TB. The study period was 25 months, including follow-up of the participants at 2 weeks and 2 months after enrolment.

### Study population

To ensure the results were generalizable, up to 2,000 adults (aged > 18 years) with presumptive TB were enrolled from four geographically diverse, high TB-burden countries—South Africa, Uganda, Peru, and India—that met the following criteria: TB incidence ≥100/100,000 population/year; 2. HIV prevalence; 3. ongoing community transmission of COVID-19; and 4. sufficient laboratory and clinical infrastructure to support participant recruitment.

#### Inclusion criteria

Participants were included in the study if they met the following criteria:

- Adult aged ≥18 years.
- Able to provide written informed consent.
- Self-reported at least one or more symptoms suggestive of pulmonary TB, such as cough ≥2 weeks, fever, night sweats, or unintended weight-loss.
- Willing to return for a day 2 visit and provide oral swab samples for biobanking.

#### Exclusion criteria

Participants were excluded from the study if:

- They had started tuberculosis preventive therapy within the past 6 months or anti-TB treatment within 60 days prior to enrolment (to avoid false-positive results due to detection of residual DNA from dead bacteria)
- They were unable to provide >3 mL of sputum or nasopharyngeal and tongue swabs on either Day 1 or 2, or before starting the third dose of anti-TB treatment.

Participants who provided consent and were enrolled but did not provide a 3 mL sputum specimen, nasopharyngeal swab, and tongue swab on either Day 1 or Day 2 were removed from the study as an early exclusion. The investigator also maintained a screening log to record details of all participants who were screened and to confirm eligibility or record reasons for screening failure. The identified eligible and consented adults were prospectively enrolled. Clinical and laboratory assessments for each participant were recorded in a clinical case report form (CRF), which was uploaded electronically on a regular basis.

### Study procedures

**Figure 1:**
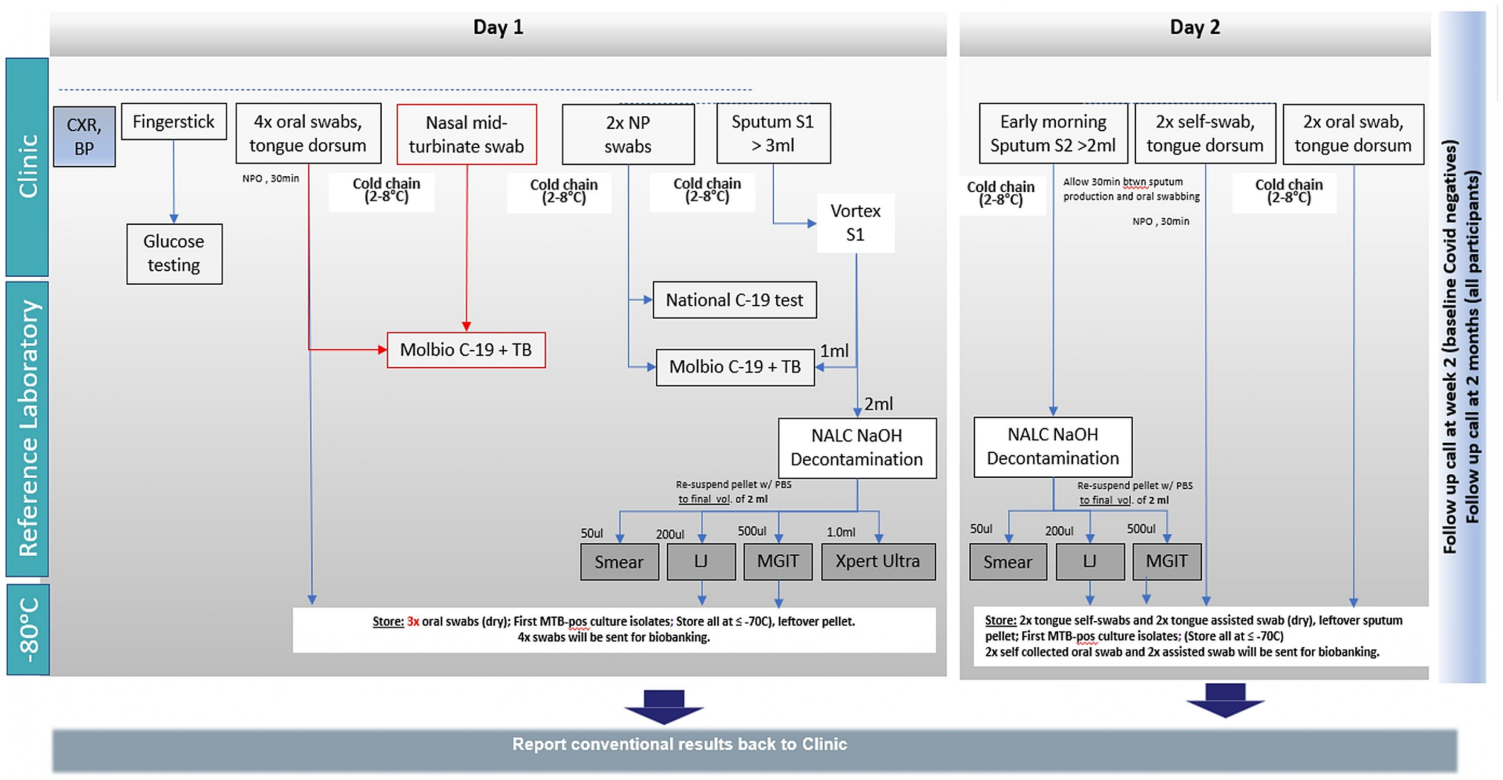
Schedule of assessments for sample type and test. BP, Blood pressure; CXR, Chest x-ray; LJ, Lowenstein-Jensen (solid culture); MGIT, Mycobacteria growth indicator tube (liquid culture); NP, Nasopharyngeal; NPO, Nil per oral; TB, Tuberculosis.

The study procedures and their timing are summarized in the schedule of assessments (Tables 2, 3; Figure 1). Protocol waivers or exemptions were not permitted.

**Table 2:** Schedule of assessments: sample types and tests performed.

Procedure	Enrolment		Follow-up		Notes
	Day 1	Day 2	2 weeks	2 months	
Inclusion and exclusion criteria	X				
Informed consent	X				
Clinical questionnaire and BP	X				
Chest X-ray	X				
Fingerstick blood (for glucose)	X				
Nasopharyngeal swab sampling	X				1x Provider-sampled NP swabs for COVID-19 PCR test and 1x for Truenat MTB Ultima/COVID-19 testing
Tongue swab sampling	X^a^	X^b^			4x provider-sampled tongue swabs for biobanking 2x self-collected and 2x provider-collected tongue swabs for biobanking
Sputum collection and testing (Truenat MTB Ultima/COVID-19, smear, LJ, MGIT, and Ultra)	X^c^	X^d^			One spot sputum for Truenat MTB Ultima/COVID-19, smear, LJ, MGIT, and Ultra One early morning sputum for smear, LJ, and MGIT
Follow-up via phone			X^e^	X^f^	Participants who tested negative for COVID-19 at enrolment All participants
AE/SAE review	X	X	X	X	

**Table 3:** Schedule of assessments: sample types and tests performed.

sample type	Name of tests
Sputum	Xpert Ultra
	Smear microscopy
	LJ culture
	MGIT culture
	Truenat™ MTB Ultima/COVID-19
Fingerstick Blood	Blood Glucose
Swabs (Nasopharyngeal)	Truenat™ MTB Ultima/COVID-19
	RT-PCR
Swabs (Oral)	Future research
NP frozen Biobanked (India)	Truenat™ MTB Ultima/COVID-19

#### Enrolment

The identified participants were prospectively enrolled in the study and recorded in the identification log. Clinical and laboratory assessments for each participant were recorded in a clinical case report form (CRF), which was uploaded electronically on a regular basis.

The participants were requested to visit the facility on two separate days for various sampling and investigations. On Day 2, a questionnaire assessing the ease of self-sampling was also administered. Procedures conducted as part of routine clinical management were utilized for screening or baseline purposes, provided they were performed within the time frame defined in the schedule of assessments.

#### Specimen collection and handling

##### Day 1

Specimens were obtained, and activities were conducted as outlined in the schedule of activities (Figure 1), including the following:

- Fingerpick for the blood glucose test.
- A 3 mL sputum split for the index test (1 mL raw sputum).

- Reference testing (2 mL sputum decontaminated) (smear, LJ, MGIT, Xpert Ultra).
- Four oral swabs (tongue dorsum) collected by the healthcare worker for biobanking.
  - Two nasopharyngeal swabs collected by the healthcare worker for the index test and COVID-19 RT-PCR testing.
- One mid-turbinate nasal swab collected by the healthcare worker for the index test.

Blood pressure measurement and digital chest X-ray examination were performed for all participants. Identifiable details from the X-ray images were removed, and, where possible, the images were stored in a databank for future studies. A sputum collection container was provided to the participants to collect a morning sputum sample on Day 2.

##### Day 2

A questionnaire assessing the ease of self-sampling was administered.

The following samples were collected within 7 days of enrolment, provided the participant had received no more than two doses of anti-TB treatment:

- ≥2 mL early morning sputum for TB reference standard testing (smear, LJ, MGIT)
- 2x tongue swabs, self-collected by the participant under observation by a healthcare worker at the clinic
- 2 x tongue swab collected directly by the healthcare worker

In the event that a sufficient volume of Day 1 sputum was not available for the Truenat MTB Ultima/COVID-19 assay, an attempt was made to perform the index test using the early morning sputum collected on Day 2 and to collect the nasopharyngeal swab on Day 2 instead of Day 1.

All oral swab samples and first positive culture isolates were stored at −70°C in a freezer equipped with an alarm system and backup power supply. Temperature logs were maintained for quality control. Details of sample transport, packaging, and documentation are provided in Supplementary material 1.

All procedures were performed by trained personnel under the required biosafety precautions. Procedures conducted as part of routine clinical management were utilized for screening or baseline purposes, provided they were performed within the time frame defined in the schedule of assessments. In addition, the participants diagnosed with TB based on the specimen results were managed through routine TB services. The probability of adverse events (AEs) occurring to a study participant while collecting the respiratory sample was deemed to be extremely low. Nevertheless, should such an event have occurred, it was managed as described in Supplementary materials 2, 3.

### Intervention

#### Investigational product and test procedure

The investigational product (IP) assay was performed according to the manufacturer’s instructions (Supplementary material 4) for all the enrolled participants. The results of the IP assay were not used for participant management. For the sake of clarity, the Truenat MTB Plus/COVID-19 multiplex assay has been rebranded by the manufacturer as Truenat MTB Ultima/COVID-19. Both tests are identical in formulation. Multiple kit lots were provided to each site to reduce any risk of possible batch effects.

The Truenat MTB Ultima/COVID-19 is a disposable, room-temperature stable, chip-based real-time duplex two-step assay, with separate DNA extraction followed by RT-PCR testing. It is based on the detection of the *IS6110* and *IS1081* genes for MTB, in combination with the *RdRP* and *Orf1a* targets for COVID-19. The same Truenat MTB Ultima/COVID-19 multiplex assay was used on a combination of tongue and nasal swabs for TB/COVID-19 detection. Nucleic acids from the samples were first extracted using the Trueprep AUTO/AUTO v2 sample prep device and the Trueprep AUTO/AUTO v2 universal sample prep kit. A total of six microlitres of the purified nucleic acids were added into a microtube containing freeze-dried PCR reagents and incubated for 30–60 s, then 6 μL of the clear suspension was pipetted out and dispensed into the reaction well of the Truenat MTB Ultima/COVID-19 chip. The chip was then inserted into the Truelab real-time micro PCR analyzer. In the case of SARS-CoV-2, the RNA was first converted into complementary DNA (cDNA) by the reverse transcriptase enzyme, and further thermal cycling then took place. At the end of the test run, separate MTB and SARS-CoV2 results of “DETECTED” or “NOT DETECTED” were displayed. A semi-quantitative result was also displayed in positive cases. Based on the detection of the internal positive control, the validity of the test run was indicated.

Each study site maintained an up-to-date inventory of the study materials, which were stored in a secure, environmentally controlled, and monitored (manual or automated) area, in accordance with labeled storage conditions. Access was limited to authorized staff. Quality control checks of the IP during transport, storage, and disposal were maintained following the study manual (Supplementary materials 5–8). New lots were used only after successfully passing qualitychecks. The sites maintained records of IP receipt, reconciliation, and final disposition.

#### Reference standard tests for TB diagnosis

A microbiological reference standard (MRS) was used for TB detection. As sputum smear examination for acid-fast bacilli can yield false-positive results—either due to other mycobacteria or prior treatment—and may be negative on Xpert Ultra or culture. Thus, we did not include sputum smear examination in the MRS tests.

Only participants with MRS + or MRS– results were evaluated. Participants with only contaminated culture results or non-determinate Xpert Ultra results were excluded from the primary analysis.

#### Case definitions for TB

Confirmed TB case: A participant with a positive result on one or more of the MRS tests: MGIT culture, LJ culture (MTBC confirmed), or Xpert Ultra on sputum.

No TB: A participant for whom all MRS tests were negative, with at least one test result confirmed as negative.

#### Reference standard tests for COVID-19 diagnosis

The reference test for COVID-19 was a country-approved RT-PCR assay. Participants with invalid test results were excluded from the primary study analysis. Error, invalid, and indeterminate rates of the assay were reported separately.

Case definitions for COVID-19: A valid positive result on the country-approved RT-PCR test.

No COVID-19: A valid negative result on the country-approved RT-PCR test.

### Follow-up

Follow-up was conducted telephonically at 2 weeks post-enrolment for the participants who tested negative for COVID-19 at baseline to determine any change in their COVID-19 status. This was intended to account for the participants who might have been tested during the early phase of infection (i.e., false negatives) but subsequently developed symptoms or retested positive for COVID-19. Similarly, a telephonic follow-up was conducted at 2 months post-enrolment for all participants (1) to determine if the participants diagnosed with confirmed TB at enrolment were linked to treatment and (2) to assess if any new TB diagnoses were made. The follow-up data were used for additional sensitivity analyses, which are described in more detail in the statistical analysis plan (SAP). Before a participant was deemed lost to follow-up, the investigator made every effort to regain contact with the participant. These contact attempts were documented in the participant’s medical record. A Participant who was unreachable at the 2-week follow-up was not considered lost to follow-up. However, if the participant remained unreachable after three attempts at different time points within the 2-month follow-up window, they were considered lost to follow-up. The site attempted to contact the participant and rescheduled the missed visit as soon as possible. The participant was counseled on the importance of maintaining the assigned visit schedule, and it was ascertained whether the participant wished to and/or should continue in the study.

### Participant discontinuation/withdrawal from the study

A participant could be withdrawn at any time at the discretion of the investigator for safety, behavioral, compliance, or administrative reasons. If a participant withdrew consent for disclosure of future information, the investigator retained and continued to use any data collected before such withdrawal of consent. If a participant withdrew from the study, they could request the destruction of any samples taken but not yet tested, and the investigator documented this in the site study records.

### Statistical considerations and sample size determination

Based on the diagnostic accuracy of the Truenat MTB Ultima/COVID-19 multiplex assay for TB detection, the sensitivity of the assay was expected to be 80%. The sample size calculation was performed based on this accuracy estimate, with a significance level of 5%, an expected precision of 7, and 80% power to obtain the confidence interval. Assuming a TB prevalence of 20%, the total number of participants needed to reach 270 bacteriologically confirmed TB cases was 1,480, considering ~10% lost to follow-up. Therefore, a minimum of 370 participants were enrolled per country. The study enrolled up to a total of 2,000 participants to ensure satisfactory power for exploratory objective 3.1. The analysis was performed on the defined populations, as shown in Table 4.

**Table 4:** Defined populations of the study.

Population	Description
Enrolled/Intention-to-test	Individuals successfully enrolled in the study
Evaluable/Per Protocol Population	Enrolled participants with all samples available and valid results for all tests (index and reference)
Partially Compliant Population	Participants who complied partially with the protocol, i.e., those for whom index test and reference test results are available but not in a complete form (e.g., missing one sample type result, contaminated culture)

### Statistical analysis plan

The statistical analysis plan (SAP) was developed and finalized for the analysis of each endpoint, the populations for analysis, procedures for missing and incomplete data, and others.

Only participants with valid MRS and index test results were included in the analysis of performance characteristics. Point estimates of sensitivity and specificity, with 95% confidence intervals based on Wilson’s score method, were calculated following the definitions in Table 5.

**Table 5:** Confusion matrix: definition of the test results and performance metrics.

	Reference standard classification			
Case prediction		Positive	Negative	Total
	Predicted positive	a	b	(a + b)
	Predicted negative	c	d	(c + d)
	Total	(a + c)	(b + d)	(a + b + c + d)

a = True Positives; b = False Positives; c = False Negatives; d = True Negatives; Sensitivity = a / (a + c); Specificity = d / (b + d).

### Ethical and regulatory considerations

The study was conducted in accordance with the protocol and the consensus ethical principles derived from the Declaration of Helsinki, Good Clinical Practice guidelines, and applicable laws and regulations. The protocol, protocol amendments, informed consent form (ICF), and other relevant documents were submitted to the institutional review board/ethics committee (IRB/IEC) at each site, following country-specific regulatory processes for approval before the trial was initiated. Written informed consent was obtained from potential participants by the investigator after explaining the nature of the study. Evidence showed that written informed consent was obtained before the participants were enrolled in the study, and ample time was given to the participants to provide consent. Illiterate participants provided a thumbprint on the ICF, along with the signature and date of an impartial witness. The ICF also explained the use of remaining respiratory samples for research and the storage of X-rays in a databank. The participants were informed that they were free to refuse participation, could withdraw from the study at any time for any reason, and could request the destruction of samples taken but not tested.

### Data and management plan

#### Record keeping, data handling, and data protection

The participants were assigned unique identifiers, which were used on all records. All clinical and laboratory data were checked for quality and protocol compliance. The participant data consisted of medical history, clinical examination, laboratory testing, and follow-up information. Observations from the clinical examination were recorded directly onto a paper CRF. Whenever it was possible, an electronic CRF (direct data capture) was used to facilitate data entry by non-clinical and non-laboratory staff. In case this was not feasible, a paper CRF was used, mirroring the design of the electronic CRF. The paper CRF consisted of some data that had been entered directly (e.g., source data) and some data that had been transcribed from other sources, such as the X-ray report. Any source data directly entered into the paper CRF were signed and dated by the person who generated the data.

Other unidentified source data were described in the Site Initiation Visit Report. Laboratory staff authorized by the PI were trained and given unique passwords to enter laboratory data, originally recorded in laboratory notebooks, directly into the electronic CRF, thereby avoiding time-consuming transcription onto a paper CRF first. These source data were entered and verified in the online clinical studies platform (OpenClinica Enterprise Edition version 4.0). The sites were provided with individual password-protected accounts to access OpenClinica, following a training session. The investigator verified the accuracy and correctness of the data entries by electronically signing the CRF. Any discrepancies from the source documents were explained. The investigator filed and retained the source documents and participant records (Table 6) for 10 years after study completion. The source data were required to be attributable, legible, accurate, and complete, and any changes to the source data had to be traceable. The investigator permitted study-related monitoring, audits, IRB/IEC reviews, and regulatory agency inspections and provided direct access to the participant’s medical records and source documents used for this study. An overview of the tasks and related documents is presented in Supplementary material 9.

**Table 6:** Source data definition and record.

Type of source data	Original place of entry
Demographics	Electronic or paper CRFs
Medical history	Electronic or paper CRFs
Usability questionnaire	Electronic or paper CRFs
Specimen collecting time and date	Field nurse records
Chest X-ray	X-ray report
Laboratory results	Lab notebook / lab CRFs
Truenat Molbio TB/COVID-19 results	Electronic, transcribed to OC CRF

#### Quality management and quality assurance

Quality control activities, protocol training, use of standard operating procedures, and capacity building were provided to each site’s staff by the study monitors. A laboratory manual describing all sample testing procedures was provided to each site prior to the commencement of the study. The sites performed quality control checks at each stage of data generation and handling to ensure that all data were reliable. The study monitors performed risk-based monitoring of source data review and verification to confirm that the data entered into the CRF by authorized site personnel were accurate, complete, and verifiable against the source documents. A data monitoring committee (DMC) was not applicable to this study due to the low-risk nature of the study.

#### Safety and incident reporting

As a diagnostic accuracy study, the probability of an adverse event (AE) or severe AE occurring in a study participant to be associated with the IP was extremely low. Therefore, safety reporting was limited in scope to events associated with sample collection and those occurring at participating laboratories using the IP. For the purposes of this study, only fatal severe AEs linked to the study procedures or medical device incidents meeting the definition of an AE or severe AE were reported (Supplementary material 2).

Medical device incidents or malfunctions that resulted in an incident were detected, documented, and reported within 24 h during the study. The definition and method of documenting medical device incidents are provided in Supplementary material 3. The medical device incident report form was sent to the manufacturer within 24 h. The investigator complied with the applicable local regulatory requirements related to the reporting of incidents to the IRB/IEC.

## Results

Participant clinical and laboratory data were recorded on paper CRFs and compiled in an online clinical studies platform. The data were analyzed using appropriate statistical tests. The participants with valid MRS and index test results were included in the analysis of performance characteristics. Point estimates of sensitivity and specificity, with 95% confidence intervals based on Wilson’s score method, were calculated. Estimates of prevalence were calculated as the ratio of the number of participants who tested positive to the total number of participants. Planned interim analysis was performed for monitoring and quality assurance purposes and did not require a database lock/soft lock.

## Discussion

While various interventions are collectively needed to achieve TB elimination, scaling up access to and use of sensitive, rapid molecular diagnostics remains crucial to identifying the ‘missing millions’ of TB cases. Moreover, there is a need to identify alternative sample collection methods, especially for individuals with weak cough, people living with HIV, younger children, and those with sputum paucibacillary TB. In addition, as sputum collection is associated with infectious aerosol production, which is problematic in the context of the COVID-19 pandemic, non-sputum specimens, such as oral swabs, may be beneficial.

This multicenter study represents the first large-scale evaluation of prospectively collected fresh samples from participants with symptoms suggestive of TB to assess the performance of the Truenat MTB Ultima/COVID-19 multiplex assay. The study also evaluated the performance of the same test using a combination of a tongue swab and a mid-turbinate nasal swab as an alternative sampling method. The Truenat MTB Ultima/COVID-19 assay enables decentralization and near-participant diagnosis of MTB and COVID-19 through its rapid, simple, robust, user-friendly design, offering “sample-to-result” capability even in resource-limited settings. This is achieved through a combination of lightweight, portable, mains−/battery-operated PCR analyzers, a DNA extraction device, and a room temperature-stable PCR chip and sample preparation kits. These features allow minimally trained technicians to generate results in under 1 h, even with limited infrastructure. Moreover, with these devices, PCR testing can also be initiated at the field level.

The Truenat MTB Plus assay was previously approved by the WHO based on the detection of the *IS6110* and *nrdZ* genes. With the development of the newly designed MTB Ultima/COVID-19 assay, evidence is needed to establish its performance, where the *IS6110* and *IS1081* (replacing *nrdZ*) MTB targets, in combination with the *RdRP and Orf1a* COVID-19 targets, are in a single assay. Previous laboratory evaluations using bio-banked samples from TB patients have shown comparable performance of the MTB Ultima/COVID-19 chip to the previously endorsed MTB Plus chip. The WHO and other organizations have prioritized the development of tests for pulmonary TB case detection that do not rely on sputum. Therefore, the study also evaluated the performance of the same Truenat MTB Ultima/COVID-19 assay using a combination of alternative samples, such as mid-turbinate and tongue swabs. Preliminary research shows that the use of tongue swabs can achieve up to 77.8% (95% CI 64.4–88.0) sensitivity and 100% (95% CI, 97.2–100.0) specificity, using sputum Xpert Ultra as the reference standard (17). A previous head-to-head comparison conducted in central reference laboratories suggested that the Truenat MTB Plus assay has similar performance to Xpert MTB/RIF (18).

Furthermore, given that diabetes and hypertension are known underlying risk factors for TB, COVID-19 hospitalization (19–21), and other co-morbidities, we simultaneously offered optional blood glucose testing (fingerprick) and optional blood pressure measurement to all enrolled participants as part of an integrated care and health screening service. This also helped us better characterize the study population. At-risk participants were provided with an explanatory referral form and instructions for seeking further evaluation and treatment at an associated or integrated clinic.

## Methodological consideration and strength

### Index test

The risk of review bias was minimal. Interpretation of the results from the index tests did not require input from the end users as it was based on pre-defined and automatically implemented thresholds. Furthermore, the results were available and recorded before those of the reference standard. If any other rapid molecular assays were performed on the same sample, laboratory staff were instructed to record the results independently of other test results.

#### Reference standard

The results from reference standard testing were recorded blinded to the index test results, thus eliminating the risk of review bias. The presence of MTB complex was confirmed for all participants with a positive culture using the MPT64 identification test and/or Line Probe Assay. The MRS had imperfect sensitivity, which could lead to bias in the accuracy estimates of the index test, and sensitivity was overestimated when both the index test and reference standard yielded negative results in a participant with TB/COVID-19. Specificity was underestimated when the index test yielded a positive result in a participant with TB/COVID-19 who tested negative with the MRS. Therefore, telephonic follow-up helped confirm the disease status of the participants (see the follow-up section). Samples for the index test assay were collected in parallel with the samples used for reference testing to avoid any disease progression bias.

#### Handling of indeterminate results

Samples for which the index test or cultures were indeterminate or contaminated were excluded from the analyses but reported separately. Although randomization was not applicable to this study design, there remained potential bias in laboratory tests that were subjected to the operator’s interpretation, such as smear microscopy and culture. To minimize this, different operators were assigned and instructed to record results independently.

#### Participant selection

Spectrum bias was avoided by enrolling consecutive participants and using a cross-sectional study design. Enrolment was based on eligibility criteria targeting participants with clinical suspicion of TB, thus representing future target populations. Descriptive statistics on participant characteristics and estimates of diagnostic accuracy stratified by site, smear status, and HIV status further ensured the validity and generalizability of the study results. Maximizing the potential of the Truenat MTB Ultima/COVID-19 assay for improving case finding required identifying an efficient method that supports both TB and COVID-19 sampling from the same specimen. Therefore, alternative samples such as tongue swabs were collected and banked for further research purposes. The study was also designed to assess the performance of the same test using alternative sample types (a combination of mid-turbinate nasal and tongue swabs) for the simultaneous detection of TB and COVID-19.

## Conclusion

Knowledge gained from this study will benefit society by improving TB diagnosis. Study participants may directly benefit from the study because they will receive a higher standard of TB diagnostic care than what is routinely available. Given the minimal risks associated with this study and the potential benefits to both society and individuals, the benefits outweigh the aggregated risks. The data gathered from this first study on the Truenat MTB Ultima/COVID-19 assay will form part of the dossier to be submitted to WHO for review. The findings from this study will contribute to the global evidence base for the development and deployment of integrated molecular diagnostics, potentially informing the WHO guidelines and advancing efforts toward TB elimination, especially in dual-burden settings.
