# Supplementary material for: Performance evaluation of the Molbio diagnostics Truenat MTB Ultima/COVID-19 multiplex assay for TB and COVID-19 case detection among people with symptoms suggestive of tuberculosis—a study protocol for clinical trials
Source: Front Public Health. 2025 Jun 27;13:1620210. doi: 10.3389/fpubh.2025.1620210 (PMC12245902; doi:10.3389/fpubh.2025.1620210)
Supplement: Supplementary file 1 [file Data_Sheet_1.PDF]

Supplementary material 1. Details of sample collection, transport, packaging and documentation

Specimen Tracking Form

Date of collection and shipment________________________Site:____Page 1 of 1

**No.**	**PID**	**Tongue swab** *(select the applicable)*	**Nasopharyngeal swab** *(Indicate N/A if Day 2)*	**Sputum** *(select the applicable)*	**Temp (LogTag) from Clinic/hospital to Lab (^{o}C)**	**Temp (LogTag)- receipt at Lab (^{o}C)**	**Deviation/Alarm Y/N? If Yes-add info RE: temp deviation and time length**
**1**	**TB050_ _ _ _ _ _**	❑4x Day 1<br>❑4x Day2	❑ 2x Day 1<br>❑ N/A	❑Day 1 spot<br>❑Day 2 early morning			
**2**	**TB050_ _ _ _ _ _**	❑ 4x Day 1<br>❑ 4x Day 2	❑ 2x Day 1<br>❑ N/A	❑ Day 1 spot<br>❑ Day 2 early morning			
**3**	**TB050_ _ _ _ _ _**	❑ 4x Day 1<br>❑ 4x Day 2	❑ 2x Day 1<br>❑ N/A	❑ Day 1 spot<br>❑ Day 2 early morning			
**4**	**TB050_ _ _ _ _ _**	❑ 4x Day 1<br>❑ 4x Day 2	❑ 2x Day 1<br>❑ N/A	❑ Day 1 spot<br>❑ Day 2 early morning			
**5**	**TB050_ _ _ _ _ _**	❑ 4x Day 1<br>❑ 4x Day 2	❑ 2x Day 1<br>❑ N/A	❑ Day 1 spot<br>❑ Day 2 early morning			
**6**	**TB050_ _ _ _ _ _**	❑ 4x Day 1<br>❑ 4x Day 2	❑ 2x Day 1<br>❑ N/A	❑ Day 1 spot<br>❑ Day 2 early morning			
**7**	**TB050_ _ _ _ _ _**	❑ 4x Day 1<br>❑ 4x Day 2	❑ 2x Day 1<br>❑ N/A	❑ Day 1 spot<br>❑ Day 2 early morning			
**8**	**TB050_ _ _ _ _ _**	❑ 4x Day 1<br>❑ 4x Day 2	❑ 2x Day 1<br>❑ N/A	❑ Day 1 spot<br>❑ Day 2 early morning			
**9**	**TB050_ _ _ _ _ _**	❑ 4x Day 1<br>❑ 4x Day 2	❑ 2x Day 1<br>❑ N/A	❑ Day 1 spot<br>❑ Day 2 early morning			
**10**	**TB050_ _ _ _ _ _**	❑ 4x Day 1<br>❑ 4x Day 2	❑ 2x Day 1<br>❑ N/A	❑ Day 1 spot<br>❑ Day 2 early morning			

Reviewed by: ………………………………………………………………………………. Date: ……………………………………………….

Nasopharyngeal Swab Collection (for routine and index test)

Procedure

1. Preparation and Equipment

a. Wear personal protective equipment (PPE), (e.ggown, nonsterile gloves, a mask, and a face shield) as per biological risk and local requirements.

b. Prepare all necessary supplies prior to beginning the procedure.

- NP swabs and tubes for routine use
- NP swabs and Trueprep AUTO transport medium for Truenattesting
- FIND barcode labels (Day1)
- Personal protective equipment
- CRFs (tablet or paper)
- Disinfectant for tuberculosis/COVID-19 sample
- Biosafety bag and transport containers with ice packs for sample transport

c. Ask the patient to sit comfortably with the head supported by a headrest.
Because there can be some minor discomfort during the procedure, this ensures the patient does not pull away from the swab. This also allows you to have more control over where the swab is going within the nasal cavity.If a headrest is not available, then the health care worker should use the nondominant hand to support the back of the patient's head.

2. Sample collection

a. Ask the patient to blow their nose prior to the start of the procedure to clear secretions from the nasal passageways.

b. Tilt patient’s head back 70 degrees

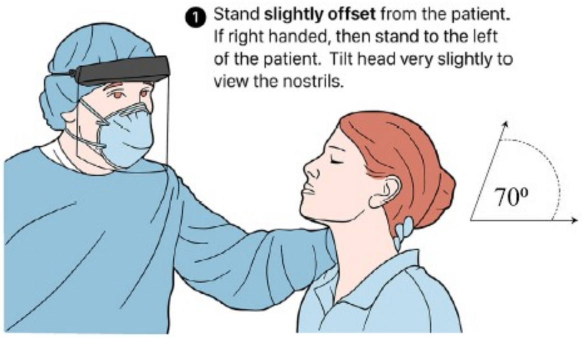

c. Gently and slowly insert the swab through the nostril as close to the floor of the nose as possible. It should be aimed both parallel to the floor of the nose and septum. If there are no obstructions from the nasal anatomy and the swab follows this trajectory, it will meet resistance once it has reached the nasopharynx.

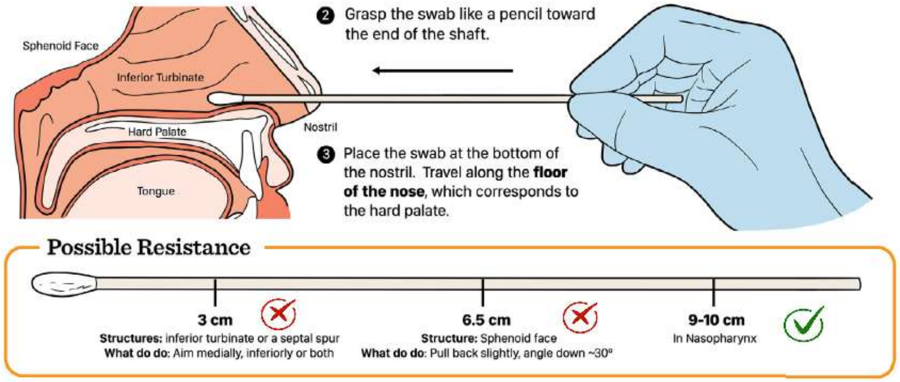

NOTE: If resistance is met almost immediately, the swab has likely hit the nasal sill. To overcome this, the swab should be aimed slightly higher to rise above this mound of tissue.

If resistance is felt at approximately 3 cm, the swab has hit the inferior turbinate. To get past the inferior turbinate, the swab should be aimed lower, medially, or both.

As the swab is advanced, the next point of potential resistance is the anterior face of the sphenoid sinus, which is found at approximately 6.5 cm. To resolve this issue, the swab should be pulled back slightly and angled down about 30 degrees to allow passage through the choana to enter the nasopharynx.

d. Once in the nasopharynx, the swab should be left in place for several seconds to absorb the secretions. It is then rotated gently, completing 5 full 360-degree rotations and then slowly withdrawn.

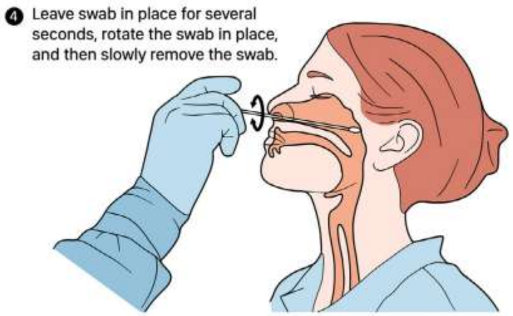

e. Place swab, tip first, into the transport tube provided. Once the tip is near the bottom, break the swab handle at the swab breakpoint by bending back and forth.
   The swab should fit in the tube comfortably so that the cap can be screwed on tightly to prevent leakage and contamination.

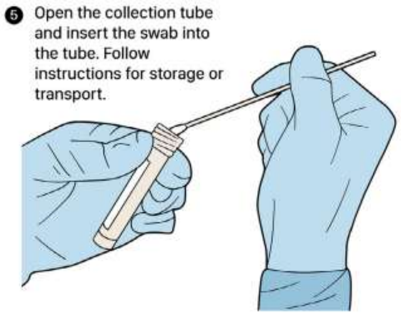

NOTE: For COVID-19 routine sample collection use the tube and swab provided in your routine care. For the MolbioTruenat MTB/COVID-19 test use the KANGJIAN disposable swabs and the TrueprepAUTO Transport Medium for Swab Specimen

2.1. Sample storage, transport and reception

- Collected samples should be stored at 2-8°C immediately after collection (cooler box with ice packs or fridge).
- Collected NP swabs should be transported to the central laboratory under temperature control (2-8°C) the same day of collection.
- Temperature, date and time of reception should be recorded in the Laboratory CRFs (corresponded to the visit day)
- Samples should be processed immediately but within max. 72 hours of collection

NOTE: Store the samples at 2-8°Cif not processed immediately

*figures taken from Kaufman AC, et al. Am J Med. 2020. 133(11):1280-1282.
