# Supplementary material for: Performance evaluation of the Molbio diagnostics Truenat MTB Ultima/COVID-19 multiplex assay for TB and COVID-19 case detection among people with symptoms suggestive of tuberculosis—a study protocol for clinical trials
Source: Front Public Health. 2025 Jun 27;13:1620210. doi: 10.3389/fpubh.2025.1620210 (PMC12245902; doi:10.3389/fpubh.2025.1620210)
Supplement: Supplementary file 2 [file Data_Sheet_2.PDF]

Supplementary Material 2: Safety Definitions and Reporting

**Adverse Event (AE) Definition**
• An AE is any unfavourable and unintended sign (including abnormal laboratory finding), symptom or disease temporally associated with the use of an investigational product, whether or not related to the investigational product.

**Serious Adverse Event (SAE) Definition:**
a. Results in death
b. Is life-threatening<br>The term 'life-threatening' in the definition of 'serious' refers to an event in which the participant was at risk of death at the time of the event. It does not refer to an event, which hypothetically might have caused death, if it were more severe.
c. Requires inpatient hospitalization or prolongation of existing hospitalization<br>In general, hospitalization signifies that the participant has been detained (usually involving at least an overnight stay) at the hospital or emergency ward for observation and/or treatment that would not have been appropriate in the physician's office or outpatient setting. Complications that occur during hospitalization are AEs. If a complication prolongs hospitalization or fulfils any other serious criteria, the event is serious. When in doubt as to whether "hospitalization" occurred or was necessary, the AE should be considered serious. Hospitalization for elective treatment of a pre-existing condition that did not worsen from baseline is not considered an AE.
d. Results in persistent disability/incapacity<br>• The term disability means a substantial disruption of a person's ability to conduct normal life functions.<br>• This definition is not intended to include experiences of relatively minor medical significance such as uncomplicated headache, nausea, vomiting, diarrhea, influenza, and accidental trauma (e.g., sprained ankle) which may interfere with or prevent everyday life functions but do not constitute a substantial disruption.
e. Is a congenital anomaly/birth defect

f. Other situations:

- Medical or scientific judgment should be exercised in deciding whether SAE reporting is appropriate in other situations such as important medical events that may not be immediately life-threatening or result in death or hospitalization but may jeopardize the participant or may require medical or surgical intervention to prevent one of the other outcomes listed in the above definition. These events should usually be considered serious.

  Examples of such events include intensive treatment in an emergency room or at home for allergic bronchospasm or convulsions that do not result in hospitalization, or development of drug dependency or drug abuse.

SAE Reporting to FIND

- The SAE Report will be sent to the FIND Head of Program and Study Manager via e-mail, marked High Priority, with a follow up call to ensure receipt.
- Initial notification via telephone does not replace the need for the investigator to complete and sign the SAE Report within the designated reporting time frames.
- Contacts for SAE reporting can be found on the front of the protocol.
