# Supplementary material for: Performance evaluation of the Molbio diagnostics Truenat MTB Ultima/COVID-19 multiplex assay for TB and COVID-19 case detection among people with symptoms suggestive of tuberculosis—a study protocol for clinical trials
Source: Front Public Health. 2025 Jun 27;13:1620210. doi: 10.3389/fpubh.2025.1620210 (PMC12245902; doi:10.3389/fpubh.2025.1620210)
Supplement: Supplementary file 3 [file Data_Sheet_3.PDF]

Supplementary Material 3: Incident Definition and Reporting

**Medical Device/IVD Incident Definition**
• A medical device incident is any malfunction or deterioration in the characteristics and/or performance of a device or IVD as well as any inadequacy in the labelling or the instructions for use which, directly or indirectly, might lead to or might have led to the death of a participant/user/other person or to a serious deterioration in his/her state of health.<br>• Not all incidents lead to death or serious deterioration in health. The non-occurrence of such a result might have been due to other fortunate circumstances or to the intervention of health care personnel.

It is sufficient that:

- An incident associated with a device happened and
- The incident was such that, if it occurred again, might lead to death or a serious deterioration in health.

A serious deterioration in state of health can include any of the following:

- Life-threatening illness
- Permanent impairment of body function or permanent damage to body structure
- Condition necessitating medical or surgical intervention to prevent one of the above
- Foetal distress, foetal death, or any congenital abnormality or birth defects

**Medical Device Incident Documenting**
• For medical device incidents fulfilling the definition above, complete the SAE Report Form.<br>• It is very important that the investigator provides his/her assessment of causality (relationship to the medical device provided by FIND) and describes any corrective or remedial actions taken to prevent recurrence of the incident.<br>• A remedial action is any action other than routine maintenance or servicing of a medical device where such action is necessary to prevent recurrence of an incident. This includes any amendment to the device design to prevent recurrence.
