# Supplementary material for: Performance evaluation of the Molbio diagnostics Truenat MTB Ultima/COVID-19 multiplex assay for TB and COVID-19 case detection among people with symptoms suggestive of tuberculosis—a study protocol for clinical trials
Source: Front Public Health. 2025 Jun 27;13:1620210. doi: 10.3389/fpubh.2025.1620210 (PMC12245902; doi:10.3389/fpubh.2025.1620210)
Supplement: Supplementary file 4 [file Data_Sheet_4.PDF]

Process flow for testing NASAL & TONGUE swab specimens
on Truenat® MTB Ultima/COVID-19
Specimen Preparation for Extraction with Trueprep® AUTO/AUTO v2 and detection
with Truelab® Uno Dx/Duo/Quattro

For Tongue Swab Specimen:

Collect the **first Tongue swab** specimen as per study procedures using a COPAN swab

⬇

Insert the swab with specimen into the Transport Medium for Swab Specimen Tube (VTM Molbio) provided and mix well by repeatedly twirling the swab in the buffer solution

⬇

Gently break the handle of the COPAN swab at the break point, leaving the swab containing the specimen in the Transport Medium for Swab Specimen Tube

⬇

Tightly close the cap of the Transport Medium for Swab Specimen Tube.
Discard* the remaining part of the swab

For Nasal Swab Specimen:

Collect Nasal swab specimen as per standard procedures using a standard nylon flock swab

⬇

Insert the swab with specimen into the Transport Medium for Swab Specimen Tube (VTM Molbio) containing a tongues swab already and mix well by repeatedly twirling the swab in the buffer solution

⬇

Gently break the handle of the nylon swab at the break point, leaving the swab containing the specimen in the Transport Medium for Swab Specimen Tube

⬇

Tightly close the cap of the Transport Medium for Swab Specimen Tube.
Discard* the remaining part of the swab

LAB PROCEDURES for Trueprep

Mix or vortex the sample (containing both Nasal and Tongue swabs) carefully by closing the cap

⬇

Transfer 500µL from the Transport Medium for Swab Specimen Tube into the Lysis Buffer tube using 1 mL Transfer pipette. Discard* the 1mL transfer pipette

⬇

Remove the Cartridge from the pouch, label it and place it on the Cartridge stand
Keep the Elute Collection Tube (ECT), ECT label and elute transfer pipette in the pouch for later use.

⬇

Transfer the entire content of the Lysis Buffer Tube to the Sample Chamber (Black Cap) of the cartridge by using the 3 mL transfer pipette

⬇

Discard* the 3 mL transfer pipette

⬇

Switch “ON” the Trueprep® AUTO v2 device. Press “EJECT” button to open and gently pull out the cartridge holder

⬇

Place the cartridge in the tray and gently push to close the cartridge holder. Press “START”

⬇

The device will beep at the end of the extraction process (20 min.) & cartridge holder will eject automatically

⬇

Gently pull out cartridge holder, remove cartridge, place it on the cartridge stand

⬇

Carefully pierce the Elute Chamber with provided elute transfer pipette and transfer the entire elute into the ECT. Discard* the transfer pipette and cartridge

⬇

Switch “ON” the Truelab® Real Time Quantitative micro PCR Analyzer

⬇

Select User ID and enter the login password

⬇

Select test profile **“MTB Ultima/COVID-19”** and enter the patient details

⬇

Select sample type **“Swab” then “Nasal swab”**

⬇

Press “START TEST” on the screen

⬇

Open the chip pouch of Truenat® MTB Plus/COVID-19
Gently take out the chip and place it on the chip tray by aligning it in the slot provided

⬇

Place the microtube containing freeze dried PCR reagents in the microtube stand

⬇

Using the filter barrier tip provided in the pouch, pipette out 6 µL of the purified nucleic acids from the Elute Collection Tube into the microtube.

⬇

Allow the mastermix to stand for 30 seconds to get a clear solution
Do not mix by tapping, shaking or reverse pipetting

⬇

Using the same micro tip, pipette out 6µL of this clear solution and load onto the centre of the white reaction well of the chip (Truenat MTB Ultima/Covid-19)
Discard* the pipette tip and mastermix tube

⬇

Press **YES** on the device screen to start the test

After completion of the reaction (≈35 mins.) press “RESULT” to go to the result screen. Observe run status (Valid/Invalid) and MTB/SARS COV-2 “DETECTED” for Positive result or “NOT DETECTED” for Negative result. The result screen would also display the bacterial/Viral load as “HIGH” (Ct<20), “MEDIUM” (20≤Ct<25), “LOW (25≤Ct<30)” (25≤Ct<30)” or “VERY LOW (Ct ≥ 30)” for positive specimen.

* Discard in freshly prepared 0.5% Sodium hypochlorite solution
Chips and Cartridges are categorized as plastic waste and these should be discarded as per biomedical waste management rules.
