# Supplementary material for: Performance evaluation of the Molbio diagnostics Truenat MTB Ultima/COVID-19 multiplex assay for TB and COVID-19 case detection among people with symptoms suggestive of tuberculosis—a study protocol for clinical trials
Source: Front Public Health. 2025 Jun 27;13:1620210. doi: 10.3389/fpubh.2025.1620210 (PMC12245902; doi:10.3389/fpubh.2025.1620210)
Supplement: Supplementary file 5 [file Data_Sheet_5.PDF]

IP Receipt Log

SUMMARY SHEET

Purpose: To record the number of investigational products (IP) received.

Best practice Recommendations:

- To be completed after each IP receipt, maintained throughout the conduct of the trial
- Dateand sign the number of IP received after **each receipt.**
- Place the IP receipt log in the storage area.
- Number and date each page and maintain the original logs in the Investigator Site File.
- Store pages in reverse chronological order, with the newest pages of the log placed at the front of the section.
- At the conclusion of the study, identify the final page of the log by checking the box in the first table.
- Remove this Tool Summary Sheet before use of the log.

**IP name**	**Lot No.**	**Expiry date** (dd/mm/yyyy)	**Number of products received** (specify if box, kits, tests, …)	**Date ofreceipt** (dd/mm/yyyy)	**Storage conditions**	**Comments**	**Recipient's Signatur e & Date**

**Investigator's Name**	**Signature**	**Date**
