# Supplementary material for: Performance evaluation of the Molbio diagnostics Truenat MTB Ultima/COVID-19 multiplex assay for TB and COVID-19 case detection among people with symptoms suggestive of tuberculosis—a study protocol for clinical trials
Source: Front Public Health. 2025 Jun 27;13:1620210. doi: 10.3389/fpubh.2025.1620210 (PMC12245902; doi:10.3389/fpubh.2025.1620210)
Supplement: Supplementary file 6 [file Data_Sheet_6.PDF]

IP Accountability Log **SUMMARY SHEET**

**Protocol Title (short):**	**Site (Name/Location):**
**Protocol Number:**	**Principal Investigator:**
	**Sponsor**

Purpose: To record the number of investigational products (IP) used and left as well as dispose of leftovers.

Best practice Recommendations:

- Date and initial the number of IP used and disposed after **each day.**
- Place the IP accountability log in the storage area.
- Number and date each page and maintain the original logs in the Investigator Site File.
- Store pages in reverse chronological order, with the newest pages of the log placed at the front of the section.
- At the conclusion of the study, identify the final page of the log by checking the box in the first table.
- Remove this Tool Summary Sheet before use of the log.

IP or Supply or Equipment Name	Lot No.	Expiry date (dd/mm/yyyy)	Date of receipt (dd/mm/yyyy)	Number of IP/Supply/Equipment				Comments	Recorder's Initials & Date
				Received	Used	Disposed	Remaining		
