# Supplementary material for: Performance evaluation of the Molbio diagnostics Truenat MTB Ultima/COVID-19 multiplex assay for TB and COVID-19 case detection among people with symptoms suggestive of tuberculosis—a study protocol for clinical trials
Source: Front Public Health. 2025 Jun 27;13:1620210. doi: 10.3389/fpubh.2025.1620210 (PMC12245902; doi:10.3389/fpubh.2025.1620210)
Supplement: Supplementary file 7 [file Data_Sheet_7.PDF]

Storage of investigational material

What shall I do upon receipt?

1. Store all reagents and instruments according to instructions in table
2. Confirm if lab temperature record is available for storage
3. Perform an inventory and cross check versus the invoice
4. Maintain the IP accountability log on a regular basis indication how many tests were used of which lot, when, by whom etc. See the logs provided in the Trial manual

**Product**	**Storage Temperature**	**Additional details**
Trueprep AUTO v2 Universal Cartridge Based Sample Prep Device	Environment temperature (15° to 45ºC), Relative humidity (10-90%)	The Truenat instrumentsare donated to the partner.
Truepet 6µl Precision Micropipette	Environment temperature (15° to 45ºC)	
Truenat Covid-19 kit	2º to 30ºC, avoid exposure to light	Stable for 2 years from date of manufacture under recommended storage conditions.
Truenat MTB+C19 Combined chip	2º to 30ºC, avoid exposure to light	Stable for 2 years from date of manufacture under recommended storage conditions.
Trueprep AUTO Transport medium for Swab Specimen	2º to 40ºC, avoid exposure to light	Stable for 2 years from date of manufacture under recommended storage conditions.
Trueprep AUTO Sputum Liquefaction Reagent 50T	2º to 40ºC, avoid exposure to light	Stable for 2 years from date of manufacture under recommended storage conditions.
Trueprep AUTO Sample Pre-treatment Pack	2º to 40ºC, avoid exposure to light	Stable for 2 years from date of manufacture under recommended storage conditions.
Truenat auto v2 Universal cartridge-based sample prep kit	2º to 40ºC, avoid exposure to light	Stable for 2 years from date of manufacture under recommended storage conditions.
