# Supplementary material for: Performance evaluation of the Molbio diagnostics Truenat MTB Ultima/COVID-19 multiplex assay for TB and COVID-19 case detection among people with symptoms suggestive of tuberculosis—a study protocol for clinical trials
Source: Front Public Health. 2025 Jun 27;13:1620210. doi: 10.3389/fpubh.2025.1620210 (PMC12245902; doi:10.3389/fpubh.2025.1620210)
Supplement: Supplementary file 8 [file Data_Sheet_8.PDF]

IP Return and Destruction Form **SUMMARY SHEET**

Purpose: To record the number of investigational products (IP) returned, destroyed, or shipped to another site.

Best practice Recommendations:

- To be completed when the IP is returned, destroyed or shipped to other site at the end of the trial or as and when required
- After each is completed, PI needs to sign and date.
- Number and date each page and maintain the original logs in the Investigator Site File.
- Store pages in reverse chronological order, with the newest pages of the log placed at the front of the section.
- At the conclusion of the study, identify the final page of the log by checking the box in the first table.
- Remove this Tool Summary Sheet before use of the log

IP or Supply or Equipment N[illegible]	Lot Number	Expiry Date	Quantity	Comments

Tick if last page [ ]

**Returned to:** Manufacturer ☐	**Destroyed at:**<br>Investigator's site* ☐Other site* ☐	**Shipped to another site***
Location:	Location:	Location:
Quantity:	Quantity:	Quantity:
By (Name of Person):	By (Name of Person)::	By (Name of Person):
Date returned:	Date Destroyed:	Date Shipped:

**Investigator's Name**	**Signature**	**Date**

Tick if last page [ ]
