# Supplementary material for: Performance evaluation of the Molbio diagnostics Truenat MTB Ultima/COVID-19 multiplex assay for TB and COVID-19 case detection among people with symptoms suggestive of tuberculosis—a study protocol for clinical trials
Source: Front Public Health. 2025 Jun 27;13:1620210. doi: 10.3389/fpubh.2025.1620210 (PMC12245902; doi:10.3389/fpubh.2025.1620210)
Supplement: Supplementary file 9 [file Data_Sheet_9.PDF]

Overview of Tasks and Related Documents

	Tasks	Forms
Clinic	Participant screening and enrolment	Screening log
	Eligibility	CRF on Eligibility
	Enrolment	Informed consent form, Participant Information Log, Barcode job aid
	Collect participant information	CRFs (clinical CRF) (OpenClinica)
	Blood glucose testing	CRF (blood glucose)
	Sample collection	WI on Tongue swab collection
		WI on Nasopharyngeal swab collection
		Job aid sputum collection
		CRFs on specimen collection (Day1, Day2)
	Specimen transport	WI on Logtags
	Chest X-ray	CRF on X-ray
	Organize follow-up	Follow-up list
	Follow-up	CRF 2 weeks FU (OpenClinica)
		CRF2 month FU(OpenClinica)
	Participant withdrawal	CRF Study completion (Open Clinica)
	SAE and AE reporting, protocol deviations	SAE Report form; AE report form; AE log; Protocol deviation log; note to file
Lab	Sputum split for reference and index test	WI Sputum reception and processing, Barcode job aid
	Decontamination, smear, MGIT, LJ, Ultra	WI Sputum reception and processing
	Truenat COMBO testing	Training material provided by Molbio
	Tongue swab storage for biobanking	WI on biobanking
	Storage of sputum pellet and isolates	WI storage sputum pellet and culture isolates
	Use of log tags	WI use of LogTag
	Receipt, use and storage of IP	Acknowledgement of receipt of IP form; Accountability of IP log; IP return form; IP destruction form; IP temperature log

Data management	Capture test results	WI data management
	Export data from LIMS	Variables for export
	Select participants for follow-up visits	Follow-up lists
	Data check	Data check lists
